# Experimental demonstration of ecological character displacement

**DOI:** 10.1186/1471-2148-8-34

**Published:** 2008-01-30

**Authors:** Jabus G Tyerman, Melanie Bertrand, Christine C Spencer, Michael Doebeli

**Affiliations:** 1 Dept. Zoology & Centre for Biodiversity, University of British Columbia, 6270 University Blvd., Vancouver, BC, V6T 1Z4 Canada; 2 Dept. Ecology and Evolutionary Biology, University of Toronto, 25 Harbord St., Toronto, Ontario, M5S 3G5, Canada; 3 Dept. Mathematics, University of British Columbia, 6270 University Blvd., Vancouver, BC, V6T 1Z4 Canada

## Abstract

**Background:**

The evolutionary consequences of competition are of great interest to researchers studying sympatric speciation, adaptive radiation, species coexistence and ecological assembly. Competition's role in driving evolutionary change in phenotypic distributions, and thus causing ecological character displacement, has been inferred from biogeographical data and measurements of divergent selection on a focal species in the presence of competitors. However, direct experimental demonstrations of character displacement due to competition are rare.

**Results:**

We demonstrate a causal role for competition in ecological character displacement. Using populations of the bacterium *Escherichia coli *that have adaptively diversified into ecotypes exploiting different carbon resources, we show that when interspecific competition is relaxed, phenotypic distributions converge. When we reinstate competition, phenotypic distributions diverge.

**Conclusion:**

This accordion-like dynamic provides direct experimental evidence that competition for resources can cause evolutionary shifts in resource-related characters.

## Background

When populations of different species occur in sympatry (together), they often have trait values that are more extreme than the values occurring in allopatric (isolated) populations [1]. For traits associated with resource acquisition or metabolism, this phenomenon is called ecological character displacement, to distinguish it from reproductive character displacement, which describes shifts in traits associated with reproduction. Ecological character displacement is observed in Galapagos finches [2-4], plethodontid salamanders [5], sticklebacks [2], Anolis lizards [6], and spadefoot toads [7,8], and is generally believed to be caused by resource competition. Theory [9-12] predicts that character displacement will result from competition selecting and maintaining extreme phenotypes to minimize phenotypic overlap and thus minimize interspecific competition.

Experiments also support the hypothesis that competition can select for divergence in resource-related traits. Schluter [13] measured selection in sticklebacks and demonstrated that growth rates and survival were depressed in the presence of competitors, and that selection was frequency dependent [14]; and Bolnick [15] showed that competition could generate disruptive selection regimes in natural populations of sticklebacks. However, selection is not evolution, and few studies have shown that interspecific competition for resources leads to evolutionary shifts in phenotypic distributions of resource-related traits [16]. Taper [16] demonstrated character shifts using bean weevils, however he failed to detect trade-offs associated with the observed shifts, thus the divergence may have evolved for reasons other than interspecific resource competition. Microbes have been employed to great advantage in studying the generation and sorting of adaptive variation [17-21]. Using microbes to test evolutionary hypotheses is possible because microbes evolve quickly in response to environmental conditions set and controlled by the researcher. Additionally, replicate populations can be studied in order to determine the repeatability of evolutionary response, and microbes can be stored indefinitely at -80° so that assays between ancestors and descendents can be conducted [reviewed in [22]]. MacLean et al. [18] used biolog plates to characterize diversification of *Pseudomonas *bacteria in response to resource competition. This study also demonstrated how diverse genotypes were maintained by frequency dependent interactions likely resulting from competition for resources. Similarly, Barrett et al. [20] showed that diversification of *Pseudomonas *generated imperfect generalists in response to competition for substitutable resources. These studies nicely illustrate how metabolic diversification occurs in the face of resource competition. While they show divergence in phenotype space, the phenotypes measured are not functionally linked to competitive performance in the environment experienced during evolution. Therefore, the importance of the phenotype for competition remains unclear. For example, it is not clear whether in experimental populations seeded with only two phenotypes, competition would lead to divergence, i.e. to an increase in the phenotypic distance between these two strains. Similarly, it is unclear what the effects of removing competition from other strains would be on a single focal phenotype.

**Figure 1:**
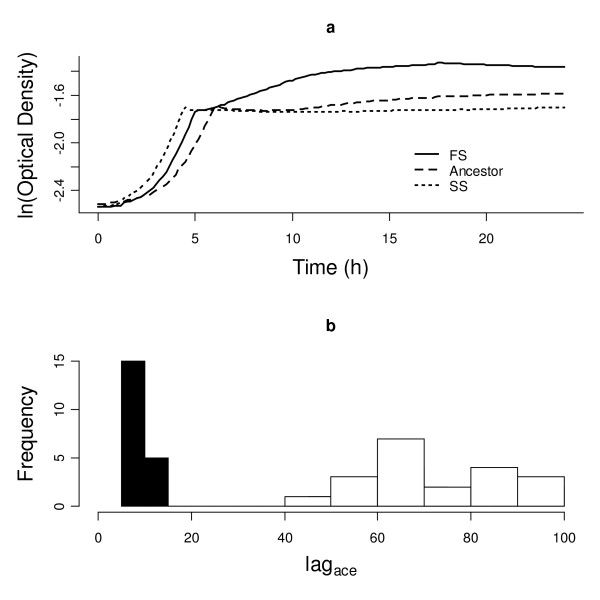
**24 h growth curves reveal resource usage differences between ecotypes**. (a) Examples of 24 h growth curves for the ancestor and derived ecotypes (Slow-switchers and Fast-switchers) from strain dst1018 after 1000 generations of evolution. (b) Histogram of *lag*_*ace *_reveals two phenotypic clusters (Fast-switchers = black and Slow-switchers = white).

Using diversified *Escherichia coli B *populations, we show in this paper that competition for resources can lead to phenotypic divergence of competing strains, i.e., to ecological character displacement, and that absence of competition can lead to phenotypic convergence. We evolved *E. coli *for 1000 generations in liquid batch cultures with glucose and acetate as sole carbon resources. Ten replicate populations diversified into cultures consisting of two ecotypes that specialized on glucose or acetate [See Additional file 1]. When *E. coli *grows in batch culture, glucose is consumed first, followed by acetate [23]. This generates a two-phase (i.e., diauxic) growth profile within a single 24 h batch cycle (Figure 1a). Diauxic growth profiles reveal how bacteria consume one resource (e.g., glucose) and switch to a second resource (e.g., acetate) only when the first is exhausted. Resource exploitation can thus be described as a metabolic reaction norm [24], and different metabolic reaction norms correspond to different 24 h growth profiles.

Our evolved cultures had diversified into two ecotypes, identifiable by different 24 h growth profiles (Figure 1a). These growth profiles were assayed in the absence of competitors of the opposite ecotype and hence are not a plastic response to the presence of a competitor. Instead, they reflect genetically distinct metabolic reaction norms, because offspring clones generate similar 24 h growth profiles as parental clones from which they descend [24]. We named the two distinct ecotypes Slow-Switchers (SS) and Fast-Switchers (FS) after differences in their relative switching lags (*lag*_*ace*_) between diauxic growth phases (Figure 1b). We extracted *lag*_*ace *_and nine additional quantifiable traits from diauxic growth curve profiles. These phenotypic traits carry the signatures of different strategies for metabolizing resources and have been shaped and maintained by competition for resources [[24], See Additional file 2].

## Results & Discussion

**Figure 2:**
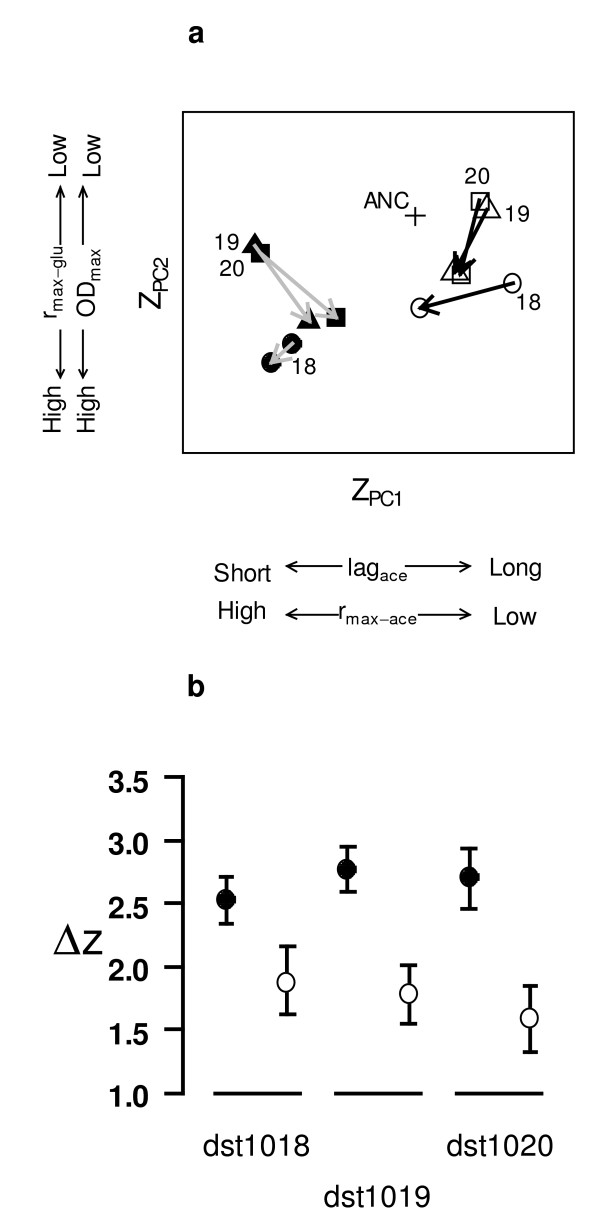
**Character displacement under competitive release**. (a) Symbols reflect mean ecotype evolutionary response from replicates (n = 20) evolved from each of three source populations (dst1018 = circles; dst1019 = triangles; dst1020 = squares), and arrows show evolutionary trajectories from sympatry to allopatry. Black symbols are FS ecotypes, white symbols are SS ecotypes. The ancestor (+) to the original evolution experiment is illustrated for comparison. Phenotypes are projected into two dimensions using the loadings from PC1 and PC2. (b) Mean distance in trait space, Δz, between ecotypes in sympatry (black) and allopatry (white) during competitive release, for replicates (n = 20) from three populations. Error bars are 95% confidence intervals.

We envisage ecotypes occupying different regions of multidimensional phenotype space, characterized by particular values of resource-related traits, z. We can measure the distance between ecotypes, Δz, under transitions from sympatry to allopatry (or vice versa), and ask whether that distance changes due to character displacement as theory predicts [9,11,12,25]. Under competitive release, i.e., moving from sympatry to allopatry, phenotypic distributions should evolve towards intermediate values and thus appear closer in phenotype space, so that the distance measured in sympatry, Δz_SYM _is larger than the distance in allopatry, Δz_ALLO _(i.e., Δz_SYM _- Δz_ALLO _> 0). We tested this prediction by evolving FS and SS ecotypes (from three populations) in isolation (i.e., under competitive release) for ~200 generations. Growth curve parameters were extracted at T1 (generation 0), corresponding to sympatry, and at T30 (generation 200), corresponding to allopatry. We measured evolutionary response as the difference in trait value (T1-T30) for each ecotype from each population. We reduced the number of traits by conducting a principle components analysis (PCA, see Additional file 3) and characterized SS and FS ecotypes in composite phenotype space (Figure 2a). We calculated the distances Δz_ALLO _and Δz_SYM_, and tested whether Δz_SYM _- Δz_ALLO _> 0. Under competitive release, we found strong support for phenotypic convergence (Figure 2b) between ecotypes from all three populations (randomization test, dst1018: P = 1.0 × 10^-6^, dst1019: P = 1.0 × 10^-6 ^and dst1020: P = 1.0 × 10^-6^). Convergence occurred primarily along the first principal component axis, with parallel shifts occurring on the remaining axes. Patterns of evolutionary response differed among populations. For example, convergence in two populations (dst1019 and dst1020) consisted of both ecotypes moving towards one another in phenotype space, but in population dst1018, convergence was due to a shift of both ecotypes in the same direction, but with SS changing to a larger extent (Figure 2a). We suspect that initial differences in position in phenotype space (dst1018 vs. dst1019 and dst1020) accounted for differences in evolutionary response of these ecotypes when released from competition.

**Figure 3:**
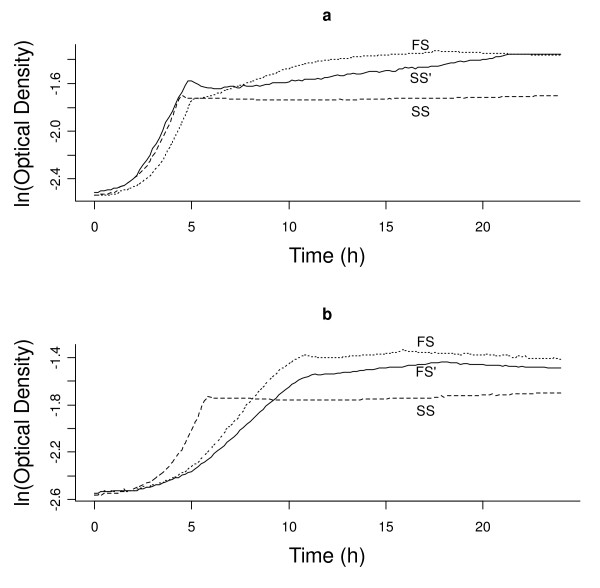
**After 200 generations (T30) of isolated evolution, "convergent" cultures (SS_ALLO _and FS_ALLO_) were assayed for intermediate genotypes**. (a) SS' ecotype derived in an ara- culture (dst1018), with FS (dotted) and SS (dashed) ecotypes shown for comparison and (b) FS' ecotype derived from an ara+ culture (dst1019) with FS (dotted) and SS (dashed) ecotypes shown for comparison.

**Figure 4:**
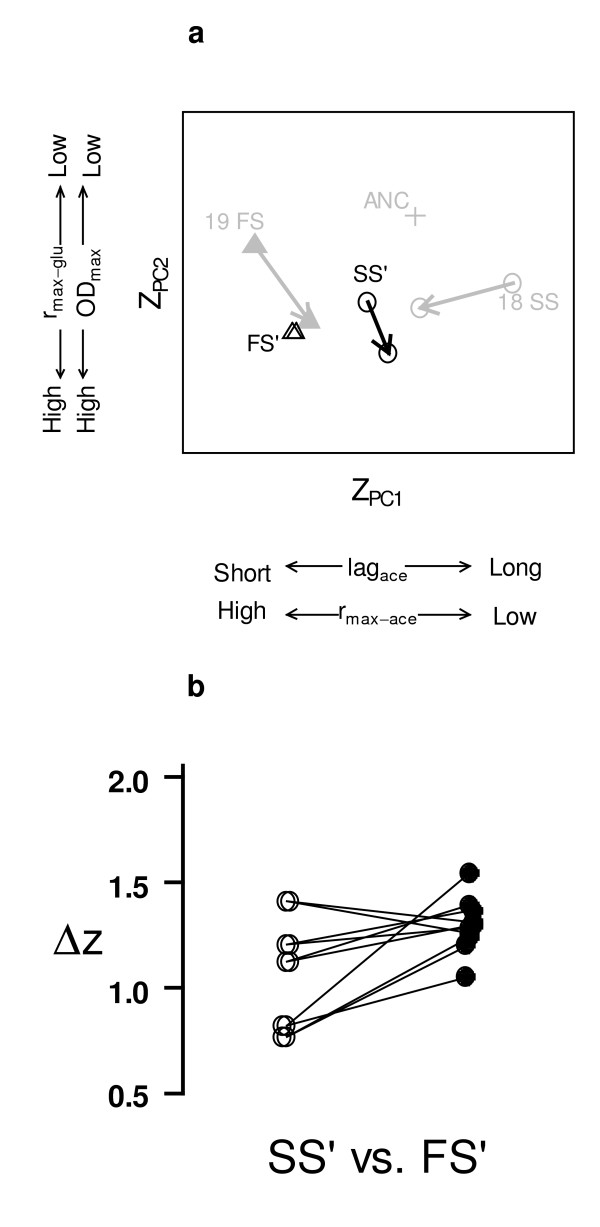
**Character displacement after competition was induced between intermediate ecotypes (SS vs. FS)**. (a) Phenotypes are projected and scaled as in Figure 2, and gray symbols and arrows illustrate the evolutionary trajectories that occurred during "competitive release" (first phase of study) in the relevant populations for comparison (see Figure 2a). Mean ecotype trajectories for SS' (circles) and FS' (triangles) from allopatry to sympatry. The black arrow shows the mean evolutionary trajectory of SS'-derived genotypes during competition, while the FS'-derived genotypes did not change substantially. (b) Distance in trait space, Δz, between pairs of SS' and FS' competitors in sympatry (white) and allopatry (black).

Next, we investigated whether adding competition would induce phenotypic divergence. For this we selected intermediate, convergent genotypes (SS' and FS'), which we isolated from T30 cultures (Figure 3, see Methods for further description). We competed SS' vs. FS' for 200 generations, after which the frequency of FS'-derived genotypes was <0.1% in 4 of the 10 competition replicates, suggesting that SS'-derived ecotypes were often able to competitively exclude FS'-derived ecotypes. Thus, we isolated genotypes derived from SS' (SS_SYM_) or FS' (FS_SYM_) from an earlier time point (generation 100, when FS was still present in an appreciable frequency in all cultures), and calculated the mean growth-curve parameters for SS_SYM _and FS_SYM_. We projected these parameters using the same composite trait space characterized during competitive release (Figure 4a). Thus, we explicitly tested whether competition could induce evolutionary divergence by directly reversing the changes that occurred during competitive release. Indeed we found that competition induced divergence (t = 2.73, df = 9, p < 0.02, Figure 4b).

Interestingly, divergence did not exactly retrace the evolutionary trajectory of convergence (Figure 4a vs. Figure 2a). Both convergence under competitive release, and subsequent divergence due to competition, occurred along the first composite trait axis. However, under competitive release, both ecotypes contributed to convergence, whereas only the SS' phenotype contributed to divergence. Moreover, the magnitude of the evolutionary response during the divergent phase was smaller than during the convergent phase (Figure 4b vs. Figure 2b). This difference in magnitude may be because we assayed character displacement after 200 generations in the first phase and only 100 generations in the second phase, allowing less time for evolution. However, the difference in magnitude of evolutionary response may also have ecological reasons. Schluter [2] argued that the speed of divergence during character displacement is greatest when phenotypic distance (i.e., degree of similarity) between competing species is intermediate. In particular, while very similar species experience intense competition, the speed of divergence is not expected to be high, because an increase in phenotypic distance may not substantially decrease competitive intensity. Instead, divergence becomes faster only after it has progressed considerably (See Figure 6.1 in ref [2]). Since the phenotypes we competed were rather similar (Figure 3), this effect may have delayed the response in our divergence treatments.

Finally, the evolutionary response under competition may be different because divergence may have occurred in phenotypic dimensions not captured by the composite trait space defined by the PCA analysis of the competitive release experiment. We conducted an independent PCA analysis on the data from only the divergence phase of our experiment, which yielded a different composite trait space. In this new trait space divergence is also significant, but the response is of similar magnitude to the evolutionary response initially identified (data not shown).

## Conclusion

Ecologists [2,9,11,12] continue to emphasize a causal role for competition in ecological character displacement. However, other factors, such as predation [21,26,27] can also affect adaptive processes of diversification. Grant & Grant [3] have therefore recently called for a definitive demonstration of competition's causal role in ecological character displacement. Here, we answer this call using experimental tests in bacterial populations. Our evidence for character convergence after competitive release is particularly compelling, and our work supports the trust that ecological theory [2,11,28,29] has placed on competition for resources as an important driver of character divergence.

Our study demonstrates that interspecific competition for resources can cause resource-related phenotypes to shift as expected in response to competition. The initial adaptive diversification generating SS and FS ecotypes, followed by our manipulations of interspecific competition (by removing and subsequently adding competitors) reveals competition's role in driving accordion-like shifts on distributions of resource-related phenotypes: divergence followed by convergence followed by divergence. Coexistence in the face of interspecific competition for shared resources may demand such an evolutionary response, with the exclusion of the inferior ecotype as an alternative outcome [30].

## Methods

### Description of evolved strains

Ten replicate populations of *E. coli *B were alternately initiated from two isogenic lines [31], which differed with respect to a neutral marker. The isogenic lines differed in their ability to utilize arabinose (ara+/-), which we exploited to discriminate between lineages in mixed cultures (see "Fitness assays" and "Competition induced" sections below). We followed the protocols of Lenski et al. [31] and others [24,32-36] with minor variations. We used large, loosely-covered test tubes, filled with 10 mL of Davis Minimal Salts media (DM) supplemented with 250 *μ*g/mL glucose and 575 *μ*g/mL acetate as the sole carbon sources. These resources were selected because diversification in their presence has been shown previously [24,33,36]. Cultures were incubated at 37° and vigorously shaken (250 rpm) for 24 h. Each day (i.e., after 24 ± 1 h of growth), 100 *μ*L of culture was transferred to 10 mL of fresh media (~1/100 dilution) and thus the seasonal cycle was reset. Each batch cycle yielded on average log_2_100 = 6.7 generations.

To test whether adaptation had occurred, we competed three populations (dst1018, dst1019, dst1020) against the ancestor of opposite marker type, and calculated relative fitness as done previously [31] (see below). These three populations were selected from the initial ten populations because there was a high correlation between colony morphology variation (large vs. small) and ecotype (SS vs. FS), which we exploited for purposes of identification in mixed culture assays. Fitness increased by ~14% [See Additional file 1] in all three populations. This suggests that adaptive evolution occurred over the course of 1000 generations.

By generation 1000, two discernable *E. coli *ecotypes, Fast-switching (FS) and Slow-switching (SS), were identified in all ten replicate populations [See Additional file 1], and there was extensive variation in frequency of the two ecotypes. We view the parallel emergence of diversity in each population as an indication that the divergence was adaptive [10].

To show that there is a functional (i.e., adaptive) explanation for the divergence in our *E. coli *populations, we assessed whether trade-offs in resource usage were detectable between SS and FS strains. From previous work [24,33,36] and this study, it appears that SS was functionally similar to the ancestor, while FS had diverged to exploit acetate earlier in the 24 h growth cycle (indicated by reduced *lag*_*ace*_, Figure 1b). Presumably this enhanced performance on acetate is associated with reduced performance on glucose. Such a trade-off has previously been found in diversified strains that have evolved under similar conditions [24,32]. To test for trade-offs in resource use, we competed SS and FS in environments that were skewed to having either more glucose or more acetate (see Fitness Assays below). A tradeoff would imply that in a glucose-enhanced/acetate-reduced environment, SS -having a metabolic profile geared towards efficient glucose use – would have higher fitness, while in an acetate-enhanced/glucose-reduced environment, FS – having a metabolic profile geared towards enhanced acetate use – would have higher fitness. Indeed, we found support for the hypothesis that tradeoffs in resource use underlie the maintenance of diversity in metabolic profiles (t = 4.305, p < 0.0005 [See Additional file 2]). This tradeoff in resource use strongly supports the hypothesis that resource competition was the selective cause for the divergence into SS and FS ecotypes.

### Asexual nature of our lines

*E. coli *exchange DNA via conjugation, passing plasmids between donor and recipient cells. However, *E. coli *B has no plasmids and can thus be considered asexual [31]. We ensured that the ancestral lines (rel606 and rel607) and evolved lines used in this study had no plasmids with a standard mini preparation of genomic DNA isolated from cells grown from each culture (Sigma GenElute Plasmid Miniprep Kit). No plasmids were detected in the ancestors or evolved cells.

### Fitness assays

Fitness of each evolved line was determined relative to the ancestor using competition experiments as described in [31]. Briefly, evolved cultures (mixed sample of SS and FS) from the endpoint of our evolution experiment (generation 1000) and cultures from the ancestors (both marker types) were inoculated from frozen stock into evolutionary media, and grown for 24 h. Evolved culture and ancestor (of opposite marker type) were mixed in equal proportions (by volume) and inoculated into fresh medium (~1/100 dilution) in ten replicates; plated on Tetrazolium agar with arabinose to determine densities at inoculation (T0), and then grown and transferred for two days before being plated to yield T2 densities. Relative fitness was calculated as ln(EV_T2_/EV_T0_)/ln(ANC_T2_/ANC_T0_) (modified from [31]), where EV is the density of evolved culture and ANC is the density of the ancestor (at times T0 and T2). To determine fitness of SS and FS in skewed resource environments (i.e., 90% [glucose]-10% [acetate] or 10% [glucose]-90% [acetate]), we isolated 10 SS and 10 FS genotypes from strain dst1018, inoculated them individually into fresh medium (50% [glucose] - 50% [acetate]) for 24 hours, and then arbitrarily selected pairs of SS and FS to mix in equal proportions (by volume). We inoculated ten pairs into both extreme environments. We plated T0 and T2 on tetrazolium agar plates (without arabinose) and used colony morphology (large or small colonies, see [24]) to aid us in determining the densities of both SS and FS ecotypes at each time point. We calculated relative fitness as above, substituting SS and FS for EV and ANC.

### Growth parameter extraction

Growth curves were obtained by inoculating ~1.5 *μ*L of conditioned culture into 150 *μ*L of fresh evolutionary medium (see above) in individual wells of a 96-well microplate. Microplate cultures were grown in a Biotek 808UI Optical Density reader, under similar conditions to the original evolutionary environment (37°, well shaken). Measurements consisted of optical densities (OD, 600 nm) obtained every 10 min over the course of 24 h. Data files were converted to a usable format using Microsoft Excel, and growth curve parameters were extracted with a program written in object oriented C++.

Table 1 summarizes the parameters extracted from growth curves. These were modified from [24]. The *StartTime *was extracted but not used directly in the analysis; it was used indirectly in the calculation of other variables (see Table 1). *StartTime *was the time where the OD (600 nm) of the growing culture first reached 0.08. Slopes were extracted using a moving window algorithm (i.e., linear regression through nine successive time points), and were used for the calculation of *r*_*max*_*glu*, *switching OD*, and *r*_*max*_*ace*. *OD*_*max *_and *OD*_*final *_were the maximum and final optical densities during the 24 h growth period. Means for each ecotype in Table 1 were calculated from twenty SS and twenty FS clones isolated from population dst1018.

**Table 1:** Description of parameters extracted from growth curves and summary data for SS and FS ecotypes (isolated from strain dst1018).

Parameter	Explanation	Slow-switcher (SS) Mean (95% CI)	Fast switcher (FS) Mean (95% CI)
StartTime	Time where optical density (OD) can be easily detected (OD = 0.08 at 600 nm) – not directly included in the analysis, but included in calculation of r_max_gluTP, timeToR_max_glu, SP, r_max_aceTP, and OD_max_TP.	--	--
r_max_glu	Maximum growth rate during "glucose phase" of diauxie.	0.081 (0.076–0.086)	0.083 (0.077–0.089)
r_max_gluTP	r_max_glu time point – StartTime.	23.5 (22.9–24.2)	27.6 (26.7–28.5)
SP	Switching (Time) Point from glucose to acetate phase – StartTime.	32.2 (31.8–32.7)	31.9 (31.4–32.4)
OD_SP_	OD of switching point.	0.24 (0.23–0.25)	0.25 (0.24–0.26)
Lag_ace_	Switching lag (time) from glucose to acetate growth	82.7 (76.3–89.0)	17.2 (10.4–24.0)
r_max_ace	Maximum growth rate during "acetate phase" of diauxie.	0.0020 (0.0015–0.0024)	0.028 (0.023–0.034)
r_max_aceTP	r_max_ace time point – StartTime	114.9 (108.7–121.2)	49.0 (42.2–55.9)
OD_max_	Maximum OD.	0.24 (0.23–0.25)	0.36 (0.34–0.38)
OD_max_TP	OD_max _timepoint -StartTime	49.2 (35.2–63.2)	80.5 (72.6–88.4)
OD_final_	Yield or OD at the end of the 24 hour period.	0.19 (0.19–0.20)	0.32 (0.31–0.33)

### Character Displacement experiments

#### 1) Competitive Release

We selected three of ten diversified populations for this experiment (dst1018, dst1019, dst1020). From the 1000-generation mark (maintained at -80°) we conditioned these populations in fresh evolutionary media for 24 h, and plated on tetrazolium agar to isolate genotypes. We selected 20 SS and FS genotypes (initially by colony morphology and confirmed by growth profile) from each population, and used these genotypes to initiate allopatric cultures (i.e., no interspecific competition). 1.5 μL of each culture was inoculated into a single well containing 150 *μ*L evolutionary media of a 96-well microtitre plate (~1/100 dilution). Growth conditions and protocols mirrored the evolutionary conditions, with the exception of differences in volume between test tubes (10 mL in the original evolution experiment) and microplate wells (150 *μ*L in this experiment). Separate microtitre plates were used for SS and FS cultures to prevent the possibility of cross-contamination between ecotypes. Although growth curves were measured on ecotypes grown in isolation (i.e., no interspecific competition), we assumed that initial growth parameter values (T1) had no mutations, and thus reflected the evolutionary signal of each ecotype under sympatry. This assumption is conservative, because we are actually measuring the parameters in isolation for the sympatric values to compare to later measures in allopatry. All cultures were propagated in isolation (allopatry) for ~200 generations by transferring 1/100 of the culture to fresh media in a new microplate every 24 hours for 30 days. After 30 days of evolution, the values obtained from growth curves were assumed to reflect the mean evolutionary response for each replicate to the treatment of allopatry. A detailed analysis of individual genotypes (beyond this study) is ongoing. Growth parameters from all derived cultures were log transformed.

#### 2) Competition induced

From T30 cultures generated in the first phase of this study (see above) we noted that all cultures were genetically heterogeneous (as determined by variation in growth curve profiles from isolated clones). Generally, there were 2–3 genotypes in FS_ALLO _(i.e., cultures derived from FS) and 2–5 genotypes in SS_ALLO _(i.e., cultures derived from SS). In many SS_ALLO _cultures, we noted one particular recurring genotype that had a decreased *lag*_*ace*_, here labeled SS' (Figure 3a). Similarly, in FS_ALLO _cultures, we noted one particular genotype with reduced maximum yields (*OD*_*MAX*_) in each phase of diauxic growth (relative to the ancestral FS genotype), here labeled FS' (Figure 3b). We considered these novel genotypes as intermediate between SS and FS ecotypes, and relatively convergent towards the opposite ecotype, when compared with the ecotype from which they were descended (SS' derived from SS and FS' derived from FS). A single SS-derived genotype (SS') was selected from one of the twenty dst1018 replicates, and a single FS-derived genotype (FS') was isolated from one of the dst1019 replicates. We used single genotypes for each novel ecotype because we wanted to focus on the role of competition (as opposed to extant genetic makeup of initially variable populations) in ecological character displacement. Additionally, a fully replicated design with all possible complimentary pairs of isolated novel genotypes in competition would be impractical.

We initiated ten mixed cultures of SS' vs. FS' (1:1, by volume) and inoculated these treatments into microplate wells (as above). We also inoculated pure SS' or FS' culture to determine z_ALLO _for each ecotype. We propagated the mixed cultures for 30 days (200 generations) to determine if competition would cause the SS' and FS' to diverge in resource-related phenotype space. Because the frequency of FS'-derived clones <0.1% by T30, we assayed our populations at T15 (See main text). We plated all replicate populations onto Tetrazolium agar (with arabinose) and identified descendent clones by their ara +/- status. Fourteen clones for each of SS'-derived and FS'-derived subpopulations from each replicate mixture were isolated and conditioned for 24 h before being assayed for growth curve parameters. We then calculated the mean parameter value from descendents from each ecotype from each competition replicate for statistical analysis (see below).

### Statistical analysis

In both phases of the experiment, we tested the hypothesis that competition caused character displacement such that Δz_SYM _- Δz_ALLO _> 0.

#### 1. Competitive release

We calculated the evolutionary response to competitive release (i.e., sympatry to allopatry) for each of ten traits by taking the difference in log-transformed trait values between T1 and T30. We pooled the evolutionary responses for all 120 replicates (3 source populations × 2 ecotypes × 20 replicates/population/ecotype). We conducted a PCA using the correlation matrix of the pooled response data [37]. We used only the first four principle components as they had eigenvalues > 1 [37], and accounted for > 81% of the variation in response to allopatry. [See Additional file 3 and Table 2 for a summary of the PCA]. We used the loadings from these four components and the difference data to generate independent (orthogonal) composite trait values. Thus, our ecotypes are described as points in four-dimensional phenotype space. From T1, we calculated:

**Table 2:** Summary of PCA conducted on correlation matrix of the difference data during competitive release. See Table 1 for explanation of parameters.

	PC_1_	PC2	PC3	PC4
Variation explained	31.3%	21.6%	16.2%	12.0%
Eigenvalue	1.77	1.47	1.27	1.09
				
Parameter:	PC Loadings			
*r*_*max*_*glu*	0.393	-0.411	-	-
*r*_*max*_*gluTP*	-0.262	0.261	0.307	0.468
*SP*	-	0.285	0.195	0.634
*OD*_*SP*_	0.197	-0.373	-0.382	0.448
*lag*_*ace*_	0.447	-	0.380	-
*r*_*max*_*ace*	-0.420	-0.140	0.224	-0.107
*r*_*max*_*aceTP*	0.384	0.153	0.398	0.248
*OD*_*max*_	-0.114	0.609	-	0.149
*OD*_*max*_*TP*	0.298	-	0.508	-
OD_*final*_	-0.330	-0.357	-0.329	0.269

Δz_SYM _= z_FS-SYM _- z_SS-SYM_

and from T30, we calculated:

Δz_ALLO _= z_FS-ALLO _- z_SS-ALLO_

where z is a vector in four dimensional trait space reflecting mean population values for FS or SS ecotypes in sympatry or allopatry. We analyzed the three source populations separately. We determined Δz_SYM _and Δz_ALLO _(and 95% C.I.) by randomly sampling 20 distances 1000 times from the fully permutated distance data set. We used a randomization test procedure to determine the probability of obtaining a test statistic (Δz_SYM _- Δz_ALLO_) that was ≥ observed data [38]. P values in the main text indicate the proportion of 100,000 analogous datasets created having (Δz_SYM _- Δz_ALLO _> observed data), after randomly reclassifying all distances into Δz_SYM _or Δz_ALLO _datasets.

#### 2. Competition induced

Our competition replicates comprised pairs (n = 10) of SS' and FS' derived genotypes. Thus, we used a paired t-test to determine whether Ha: Δz_SYM _- Δz_ALLO _> 0. This allowed us to quantify evolutionary response (i.e., divergence) in each replicate (i.e., Δz_SYM_) separately, so that divergence across replicates could arise even if ecotypes made different contributions to divergence in different replicates.

## Abbreviations

SS: slow-switching ecotype; FS: fast-switching ecotype; *lag*_*ace*_: acetate lag; Δz: distance in phenotypic (trait) space.

## Authors' contributions

JT conceived the experiments, conducted the experiments, analyzed the data, and wrote the manuscript. MB and CCS conducted the experiments. MD conceived the experiments and wrote the manuscript.

## Supplementary Material

Additional file 1 Figure S1. (a) Relative fitness of the ancestor (Generation 0) and three populations (Generation 1000) versus the ancestor of opposite marker type (ara+/-). The dashed horizontal line is equivalent fitness, error bars indicate 95% confidence intervals, and letters above error bars denote significantly different groups. (b) The proportion of SS (95% CI) in ten replicate populations evolved in glucose-acetate environment (populations in rank order). The dashed horizontal line represents the grand mean for all populations. Click here for file

Additional file 2 Figure S2. Competition experiments in skewed resource environments reveal that mean SS fitness is greater than mean FS fitness when [glucose] is enhanced (from 50% to 90%) and [acetate] reduced (from 50% to 10%) (left) and that mean SS fitness is lower than mean FS fitness when [glucose] is reduced and [acetate] enhanced (right). The horizontal line indicates equal fitness, and the error bars indicate 95% CI. Click here for file

Additional file 3 Figure S3. Principle component analysis (PC1 vs. PC2) on differences between sympatric and allopatric trait values for Slow-switchers (white) and Fast-switchers (black) from replicates initiated from three populations (dst1018 = circles, dst1019 = triangles, dst1020 = squares). Click here for file
