# Supplementary figures and images for: Experimental demonstration of ecological character displacement
Source: BMC Evol Biol. 2008 Jan 30;8:34. doi: 10.1186/1471-2148-8-34 (PMC2267161; doi:10.1186/1471-2148-8-34)

a

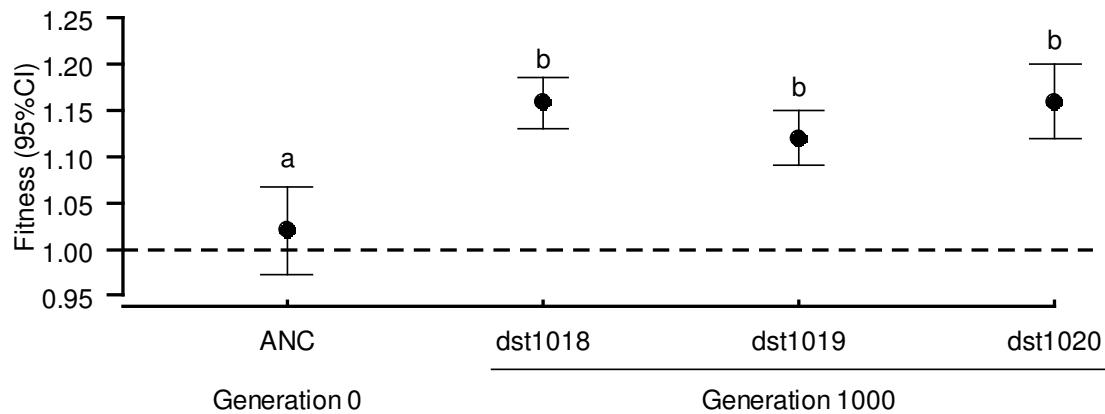

b

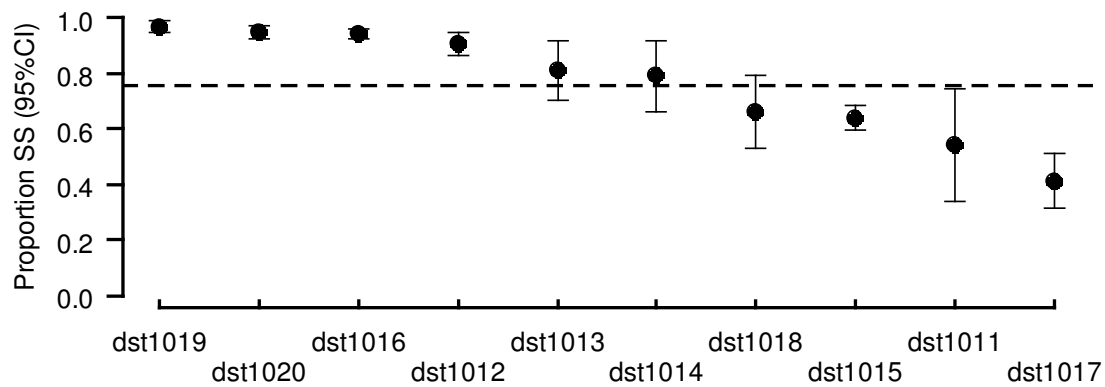

Supplement: Additional file 1 — Figure S1. (a) Relative fitness of the ancestor (Generation 0) and three populations (Generation 1000) versus the ancestor of opposite marker type (ara+/-). The dashed horizontal line is equivalent fitness, error bars indicate 95% confidence intervals, and letters above error bars denote significantly different groups. (b) The proportion of SS (95% CI) in ten replicate populations evolved in glucose-acetate environment (populations in rank order). The dashed horizontal line represents the grand mean for all populations. [file 1471-2148-8-34-S1.pdf]

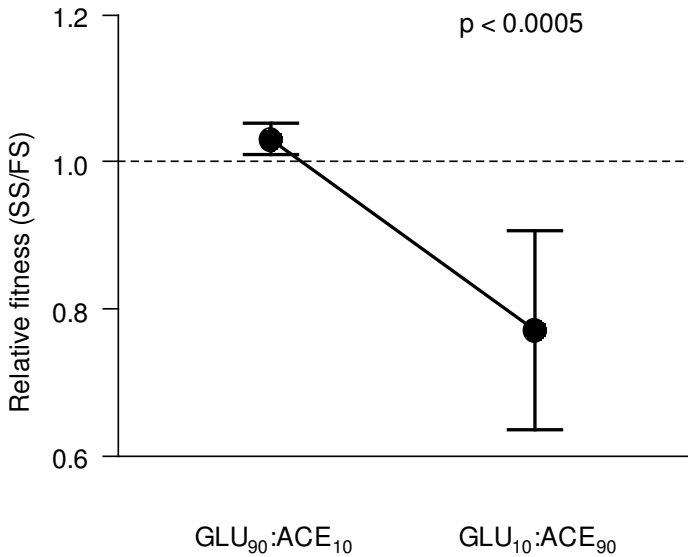

Supplement: Additional file 2 — Figure S2. Competition experiments in skewed resource environments reveal that mean SS fitness is greater than mean FS fitness when [glucose] is enhanced (from 50% to 90%) and [acetate] reduced (from 50% to 10%) (left) and that mean SS fitness is lower than mean FS fitness when [glucose] is reduced and [acetate] enhanced (right). The horizontal line indicates equal fitness, and the error bars indicate 95% CI. [file 1471-2148-8-34-S2.pdf]

PC2

PC1

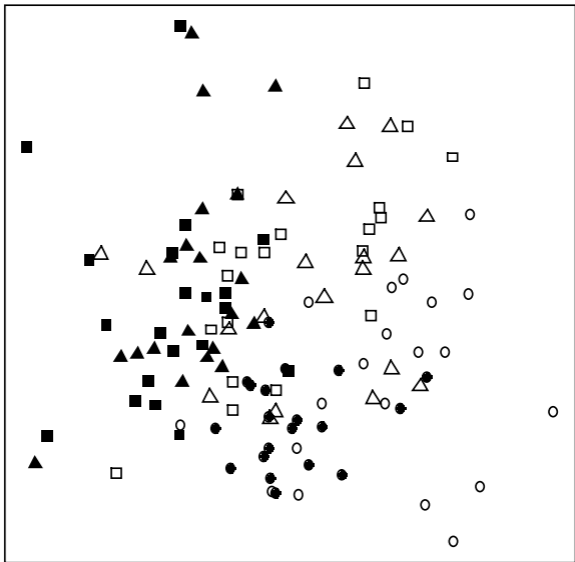

Supplement: Additional file 3 — Figure S3. Principle component analysis (PC1 vs. PC2) on differences between sympatric and allopatric trait values for Slow-switchers (white) and Fast-switchers (black) from replicates initiated from three populations (dst1018 = circles, dst1019 = triangles, dst1020 = squares). [file 1471-2148-8-34-S3.pdf]
